# Study design and parameter estimability for spatial and temporal ecological models

**DOI:** 10.1002/ece3.2618

**Published:** 2016-12-30

**Authors:** Stephanie Jane Peacock, Martin Krkošek, Mark Alun Lewis, Subhash Lele

**Affiliations:** ^1^ Ecology and Evolutionary BiologyUniversity of TorontoTorontoONCanada; ^2^ Biological SciencesUniversity of AlbertaEdmontonABCanada; ^3^ Salmon Coast Field StationSimoom SoundBCCanada; ^4^ Mathematical and Statistical SciencesUniversity of AlbertaEdmontonABCanada; ^5^ Present address: Biological SciencesUniversity of CalgaryCalgaryABCanada

**Keywords:** modeling, spatial or time series, statistics

## Abstract

The statistical tools available to ecologists are becoming increasingly sophisticated, allowing more complex, mechanistic models to be fit to ecological data. Such models have the potential to provide new insights into the processes underlying ecological patterns, but the inferences made are limited by the information in the data. Statistical nonestimability of model parameters due to insufficient information in the data is a problem too‐often ignored by ecologists employing complex models. Here, we show how a new statistical computing method called data cloning can be used to inform study design by assessing the estimability of parameters under different spatial and temporal scales of sampling. A case study of parasite transmission from farmed to wild salmon highlights that assessing the estimability of ecologically relevant parameters should be a key step when designing studies in which fitting complex mechanistic models is the end goal.

## 1 Introduction

*A model in its elegance*
*Is better than reality*
*Its graphical simplicity*
*Denotes a rare intelligence*.
*The simple graph incites the wrath*
*Of field men who, half undressed,*
*Go rushing out to start a test*
*Which culminates in aftermath*.
John McLauren Burns (1975) BioGraffiti: A Natural Selection

Models are useful tools for understanding and predicting patterns in ecological data (Hilborn & Mangel, 1997; May, 2004). The processes underlying ecological patterns are often complex, involving many interacting factors. Advances in the statistical methods commonly applied by ecologists are making it possible to fit increasingly complex models to ecological data. Examples include hierarchical models accounting for multiple sources of variability, such as state‐space models (Buckland, Newman, Thomas, & Koesters, 2004; Fleischman, Catalano, Clark, & Bernard, 2013) and mixed‐effects models (Bolker et al., 2009), and nonlinear dynamic models describing how populations change in space and/or time (e.g., Clark & Bjørnstad, 2004). Such models have helped maximize the understanding gleaned from ecological data that are often noisy and sparse. However, fitting more complex models comes with the increased risk that model parameters may not be estimable—a potential problem too‐often ignored by ecologists (Lele, 2010).

Parameter non‐estimability can result from two sources: a) structural nonidentifiability, a problem, that is, associated with the structure of the model that is being fitted, and b) practical nonidentifiability, also called nonestimability arising from the inadequacy of the particular data at hand (Campbell & Lele, 2014; Raue et al., 2009).

Structural nonidentifiability occurs when two or more parameters cannot be uniquely identified even when an infinite amount of data is available. A simple example is the inability to distinguish the magnitude of two sources of error that are additive; that is, if $Y_{i}|\mu_{i}∼N\left(\mu_{i},\;\sigma^{2}\right)$ and $\mu_{i}∼N\left(\mu ,\;\tau^{2}\right)$ , then $Y_{i}∼N\left(\mu ,\sigma^{2}+\tau^{2}\right)$, and the parameters σ^2^ and τ^2^ cannot be uniquely identified no matter how many data points are collected (Lele, 2010). This may seem obvious for this simple example, but determining structural identifiability can be difficult for more complex models (Wu, Zhu, Miao, & Perelson, 2008).

Given that the parameters of a model are structurally identifiable, they may still be nonestimable if the data are observed at the wrong points or intervals in space or time (i.e., statistical estimabilty; Campbell & Lele, 2014). Even if the model parameters are identifiable in theory and the data are collected with precision, inference may not be possible if those data do not adequately capture the process being modeled. For example, species invasions are often driven by rare long‐distance dispersal events that may not be observed without thorough sampling at the appropriate spatial scale (Clark, Lewis, McLachlan, & HilleRisLambers, 2003; Kot, Lewis, & van den Driessche, 1996). Without information on the magnitude and frequency of these dispersal events, inferring speed of population spread will be difficult or impossible. Such problems would be avoided if researchers were to consider parameter estimability along with choice of model when designing studies.

In this study, we are concerned with statistical estimability of parameters in ecological models. We show that data cloning, a new statistical tool for obtaining maximum likelihood parameter estimates using Bayesian machinery (Lele, Dennis, & Lutscher, 2007), can be used in simulation studies to determine the appropriate spatial and/or temporal scale of sampling to ensure that model parameters of interest are estimable. To illustrate this, we use data cloning to evaluate parameter estimability for an established model of parasite dispersal from point sources along a corridor (Krkošek, Lewis, Volpe, & Krkosek, 2005) under three different spatial scales of sampling. We begin with a description of the data cloning method and then introduce our case study, followed by a general discussion of how data cloning can aid in the design of ecological studies.

### 1.1 What is data cloning?

Data cloning, also known as “prior feedback” (Robert, 1993), was conceived as a way to obtain maximum likelihood parameter estimates using a Bayesian framework (Lele et al., 2007). Bayesian methods have achieved popularity among ecologists wanting to fit complex models (Ellison, 2004) due to the computational advantages of Markov Chain Monte Carlo (MCMC) for hierarchical models and the availability of free and accessible software to implement MCMC (e.g., WinBUGS (Ntzoufras, 2009) and JAGS (Plummer, 2003)). In the Bayesian approach, inference is based on the posterior distribution, which is proportional to the likelihood of the data given the model multiplied by the prior distribution of the model (Ellison, 2004). The prior is chosen by the researcher and therefore introduces a degree of subjectivity into the analysis. This can be an advantage when there is a wealth of prior information the researcher wishes to incorporate, but more often than not, such prior information is lacking in ecological studies and there is a desire for objective parameter estimates that are invariant to the choice of prior (Lele, 2010; Lele & Dennis, 2009).

Data cloning removes the influence of the prior distribution in a Bayesian analysis by raising the likelihood to some power, *K*, where *K* is the number of “clones” of the data. As *K* approaches infinity, the mean of the resulting posterior distribution approaches the maximum likelihood estimate (MLE) and the posterior variance is 1/*K* times the variance in the MLE (see Lele, Nadeem, & Schmuland, 2010 for proof). Thus, given enough clones, the posterior distribution should be invariant to the choice of prior provided the prior has nonzero probability around the highest peak of the likelihood. In practice, data cloning is carried out by running a Bayesian analysis using *K* copies of the data. An R package called dclone that integrates with existing MCMC software is available that makes data cloning easy to implement (Sólymos, 2010). This package uses the Bayesian machinery of MCMC and is thus easy to implement even for dynamical models that must be solved numerically and/or hierarchical models with latent variables or random effects. However, we note that there are alternative methods for optimizing a cloned likelihood, such as Laplace approximation (Baghishani, Rue, & Mohammadzadeh, 2012), that may be more efficient in certain cases (e.g., when using Gaussian Markov Random Fields and closely related latent structures).

One major advantage of data cloning is that the results can be used to assess the statistical estimability of parameters (Lele et al., 2010). If a parameter is estimable, then the variance in the posterior distribution should decline to zero as *K* is increased, ideally at the rate of 1/*K*. If a parameter is nonestimable, then the posterior distribution for that parameter will converge to a truncated prior distribution with nonzero variance as *K* is increased. Thus, a simple diagnostic plot of the variance in posterior distribution over *K* can be used to assess estimability. Further diagnostics have also been developed to rigorously test estimability under different prior assumptions (Campbell & Lele, 2014).

Tests of parameter estimability using data cloning can be performed on simulated data to determine whether the parameters of interest are estimable given a certain frequency of sampling in space and/or time. This is similar to the idea of a power analysis to determine the sample size required to detect an effect should one exist (Peterman, 1990; Toft & Shea, 1983), but considers the subtleties of spatial and temporal sampling intervals that can affect parameter estimability in mechanistic models. Just as a power analysis requires an estimate of variance among observations, simulations to determine estimability of model parameters may require some a priori knowledge of the spatial and/or temporal scale of patterns in the data. In the following section, we illustrate this novel approach with a case study of the spatial sampling design needed to estimate parameters in a mechanistic model of sea louse transmission from farmed to wild salmon in the narrow inlets of British Columbia, Canada (Krkošek, Lewis, et al., 2005).

## 2 Case study: Estimating sources of sea lice on wild juvenile salmon

**Figure 1:**
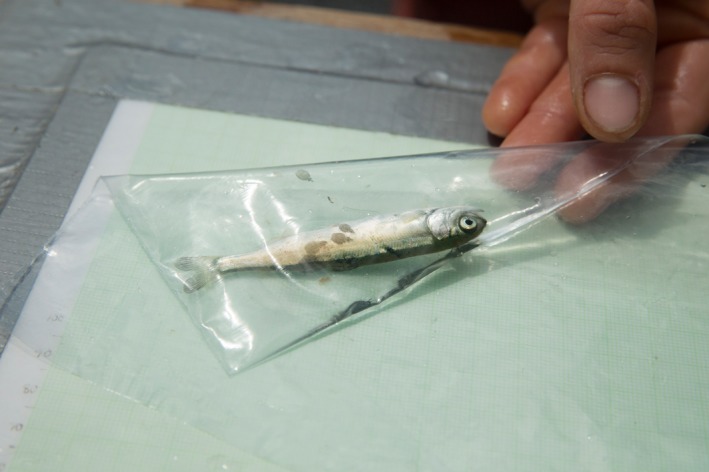
A juvenile pink salmon infected with several adult sea lice (*Lepeoptheirus salmonis*) is measured as part of a spatially intensive monitoring program in the Broughton Archipelago, Canada

Sea lice (*Lepeoptheirus salmonis* and *Caligus* spp.) are parasitic marine copepods that naturally occur on wild salmon (Figure 1), but out‐migrating juvenile salmon are normally relatively parasite‐free until they encounter adults in the open ocean. However, salmon farms have introduced a novel host population near rivers that can transmit sea lice to juvenile wild salmon when they are small and vulnerable to the impacts of the parasites (Krkošek, 2010). Due to the potential impact of sea lice on wild salmon survival, there has been considerable interest in quantifying the relative importance of salmon farms as a source of infection for juvenile wild salmon.

We consider a mechanistic model describing the infection of migrating, juvenile wild salmon with sea louse parasites from both distributed sources (e.g., adult wild salmon) and a point source at the location of a salmon farm (Krkošek, Lewis, Morton, Frazer, & Volpe, 2006; Krkošek, Lewis, et al., 2005). The model includes the advection and diffusion of free‐living sea louse larvae from the point source, yielding a spatial distribution of infectious larvae, and the attachment and development of sea lice on juvenile salmon migrating through this distribution of larvae.

### 2.1 Data

**Figure 2:**
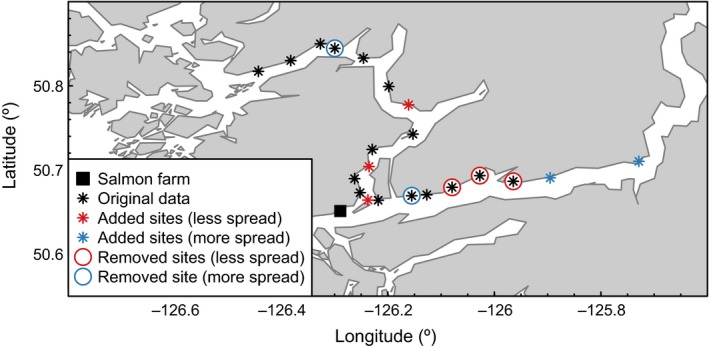
Map of study area showing the location of an active salmon farm (square) and sampling locations of juvenile wild salmon (*n *=* *16, stars). Juvenile salmon migrate along the corridor, from east (right) to west (left). We investigated the effect of more/less spatially spread sampling locations by simulating data where sample sites are moved (circled stars) to earlier locations along the migration (blue stars) or within the range of existing sample locations (red stars)

We fit the model to infection data from spatially intensive surveys of juvenile wild salmon throughout their migration. Surveys have taken place in the Broughton Archipelago, Canada (Figure 2) from 2003 to 2012. For simplicity, we focused on 2003 when there was just one active salmon farm along the migration route, thereby minimizing the number of free parameters to be estimated (see Section 2.2 and Table 1 for a summary of model parameters). The model can also be applied to the scenario where there are multiple point sources of infection (Krkošek et al., 2006; Morton, Routledge, McConnell, & Krkošek, 2010).

**Table 1:** Free parameters estimated using the sea louse transmission model of Krkošek, Lewis, et al. (2005) and described here in Equations (1), (2), (3), (4). Fixed parameters include the advection coefficient and survival/development of free‐living larvae, as given in Krkošek et al. (2006)

Parameter	Description	Equation	Prior mean^a^
*D*	Diffusion coefficient in dispersal from farm point source	Krkošek, Lewis, et al. (2005) and Krkošek, Morton, et al. (2005): Appendix A	3.5
κβ*v* ^−1^	Strength of background sources		−7.0
αβ*v* ^−1^	Strength of farm source		1.0
*s*_c_, *s*_h_	Survival of copepodid‐ and chalimus‐stage lice to the next stage	Equations (2), (3)	2.0
λ_c_	Distance travelled by juvenile salmon during the duration of the copepodid stage	Equation (1)	1.0
*L*_h_ = λ_h_/λ_c_ *L*_m_ = λ_m_/λ_c_	The distances travelled during cumulative time for development of lice to chalimus and motile stages, relative to λ_c_	Equations (2), (3)	3.0

a Mean (μ) for normal priors on log‐transformed (or logit‐transformed for *s*_c_, *s*_h_) parameters, with standard deviation σ = 0.5 for all parameters. See Supporting Information for results under different prior distributions.

The data include the number of *L. salmonis* or *C. clemensi* sea lice per wild juvenile salmon. Salmon were collected from sixteen sites that spanned the Knight Inlet—Tribune Channel migration route from 20 km before the farm location to 40 km after the farm location (dataset II‐Apr in Krkošek, Lewis, et al. (2005); Figure 2). At each site, anywhere from 100 to 258 salmon were sampled, depending on availability. Each salmon was visually inspected for sea louse parasites before being released at the location of capture (see Krkošek, Morton, & Volpe, 2005 for further details of sampling methodology).

Sea lice were classified according to their developmental stage as copepodid, chalimus, or motile. Copepodid‐ and chalimus‐stage sea lice are tethered to their host and cannot move among hosts (Boxaspen, 2006). The developmental stages of attached sea lice therefore act as biological tags that indicate the approximate time of infection. The number of sea lice on hosts in a sample can be used to infer the infection pressure at a previous point in the migration, using a mechanistic model previously published by Krkošek, Lewis, et al. (2005), Krkošek et al. (2006) and as described below in Section 2.2.

#### 2.1.1 Spatial spread of sampling

In addition to fitting the model to the original data, we also fit the model to two alternative scenarios for the sampling design to investigate how changes in the spatial spread of sampling sites affected parameter estimability. To simulate a decrease in the spatial spread of sample locations, we removed the first three sample sites from the original data (−20, −15.5, and −11.5 km from the farm; shown by red‐circled sites in Figure 2). We then added three sites at 0, 5, and 16 km (shown by red stars in Figure 2) and simulated data for these added sites (details below). The site locations were chosen to spatially distribute the sampling effort as evenly as possible within the range of the remaining original data (Figure 2). Similarly, to investigate whether a greater spatial spread in sample locations would make the key parameters of interest estimable, we removed two sites from the middle of the sampling route in the original data (−6.0 and 29.5 km; shown by blue‐circled sites in Figure 2) and added two sampling locations earlier in the migration (−30 and −40 km; shown by blue stars in Figure 2).

We simulated the number of copepodid, chalimus, and motile sea lice at the added sites by drawing Poisson random variables with expected value equal to the model prediction for the new sampling location, using parameter values from the fits to the original data. In order to facilitate comparisons among sampling designs, we used the same numbers of fish when simulating the data as was sampled at the removed sites so that the number of data points was the same among the original, less‐spread, and more‐spread datasets.

### 2.2 Model

The model follows the approach of Krkošek, Lewis, et al. (2005) and considers the migration corridor of juvenile salmon (Figure 2) as a one‐dimensional domain. Along this corridor, there is a constant ambient density of infectious sea lice from wild sources, *L*_0_(*x*) = κ. Larval sea lice also disperse according to simple advection and diffusion from a point source at a salmon farm along this migration corridor, and develop into the infectious stage, yielding a spatial distribution of infectious sea lice originating from the farm, *L*_1_(*x*) (see Krkošek, Lewis, et al., 2005 for details). The total density of infectious sea lice is therefore *L*(*x*) = κ + α *L*_1_(*x*), where α is a parameter controlling the strength of the farm source. The original model also included an additional term, *L*_2_(*x*), describing the production of larval sea lice from infected migrating juvenile salmon. However, we do not include this additional term in our modeling described here so as to keep the presentation and analysis as simple as possible. The expected number of sea lice on juvenile salmon migrating at speed *v* is proportional to the density of infectious sea lice encountered previously during their migration:

(1) $$C\left(x\right)=\frac{\beta }{v}\int_{x-\lambda_{c}}^{x}L\left(u\right)du$$

(2) $$H\left(x\right)=s_{c}\frac{\beta }{v}\int_{x-\lambda_{h}}^{x-\lambda_{c}}L\left(u\right)du$$

(3) $$M\left(x\right)=s_{h}s_{c}\frac{\beta }{v}\int_{x-\lambda_{m}}^{x-\lambda_{h}}L\left(u\right)du$$

where β is the transmission coefficient, *s*_c_ and *s*_h_ are the survival of copepodid (c) and chalimus (h) stages, λ_c_, λ_h_, and λ_m_ are the cumulative distances a salmon will travel during the developmental times of the copepodid, chalimus, and motile stages, respectively.

We calculate the likelihood of the observed number of sea lice on juvenile salmon assuming that the number of sea lice, *N*_*i*_(*x*), of stage *i* on a juvenile salmon at a given point in space, *x*, is a Poisson random variable. The likelihood of observing *j* lice of stage *i* at sampling location *x* is therefore

(4) $$P\left\{N_{i}=j;\;\lambda =I\left(x\right)\right\}=\frac{I{\left(x\right)}^{i}}{i!}e^{-I(x)}$$

where *I*(*x*) is the model‐predicted number of lice of stage *i* from Equations (1), (2), (3). The free parameters to be estimated are summarized in Table 1. Other parameters, including the advection, development, and mortality parameters controlling the dispersal of lice from farms, were fixed at previously estimated values (see Krkošek et al., 2006). The transmission coefficient, β, and migration speed, *v*, always appear together in the model as β*v* ^−1^ and thus cannot be uniquely identified (an example of structural nonidentifiability). Further, they appear only as multiples of κ or α. Therefore, estimates of parameters controlling the density of infectious larvae are in proportion to the transmission coefficient β and inversely proportional to the migration speed *v* (i.e., κβ*v* ^−1^ and αβ*v* ^−1^).

### 2.3 Data cloning

We used data cloning to estimate the free parameters in the sea louse transmission model (Table 1) and assess the estimability of these parameters. In particular, we wanted to know whether the parameters of biological interest—that is, the ambient density of infectious lice, κ, and the strength of the farm source, α—were estimable given the available data. We fit the model in a Bayesian framework using the software JAGS (Plummer, 2003), interfacing with R (R Development Core Team 2016) using the packages dclone (Sólymos, 2010) and rjags (Plummer, 2016).

We fit the model to three different datasets (in order of increasing spatial spread of sampling locations): (1) data with three sites moved to simulate less spatial spread, (2) the original data, (3) data with two sites moved to simulate more spatial spread (Figure 2). For each dataset, we assumed normal priors on the log‐ or logit‐transformed parameters (Table 1). If parameters are estimable, the maximum likelihood estimates from data cloning should be invariant to the choice of prior (Lele et al., 2007), even for priors that are far apart in their means (Campbell & Lele, 2014). To test this, we fit the model under three different prior assumptions, each with different means and standard deviations (Table S1). Each fit consisted of three independent chains initiated with parameter values drawn randomly from their prior distributions. The use of different starting points is important in the case of multimodality in the likelihood surface; estimability results may be misleading if only a single chain started near the MLE is used. Each chain was allowed 5,000 MCMC iterations for adaptation (where the JAGS software adapts the algorithm for maximum efficiency of the samplers) and a burn‐in of 40,000 iterations, using the subsequent 20,000 iterations as posterior samples. We report results for *K *=* *1 to 25 clones of the data. For each number of clones, we ran three independent MCMC chains and assessed convergence of the chains by calculating the Gelman and Rubin's convergence diagnostic ($\hat{R}$; Gelman & Rubin, 1992). We considered the chains to be well mixed and to have converged to the target distribution if $\hat{R}$ ≤ 1.1.

## 3 Results

**Figure 3:**
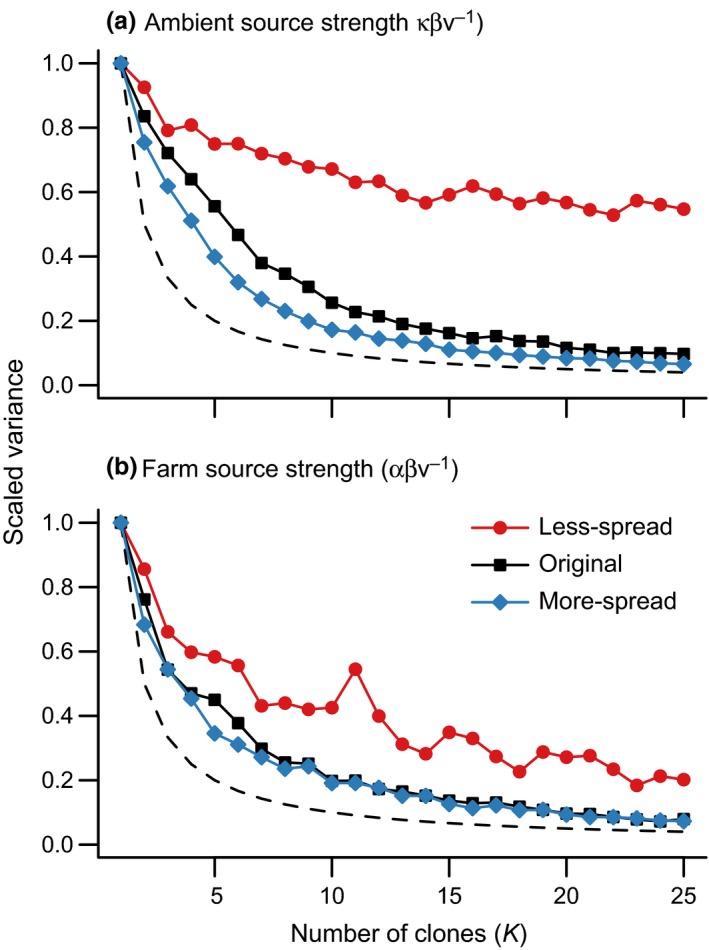
Posterior variance for (a) κ and (b) α scaled by the variance for a single clone over the number of clones (*K*) for three different data scenarios. The dashed line indicates the ideal rate of convergence to a variance of zero as *K* goes to infinity

**Figure 4:**
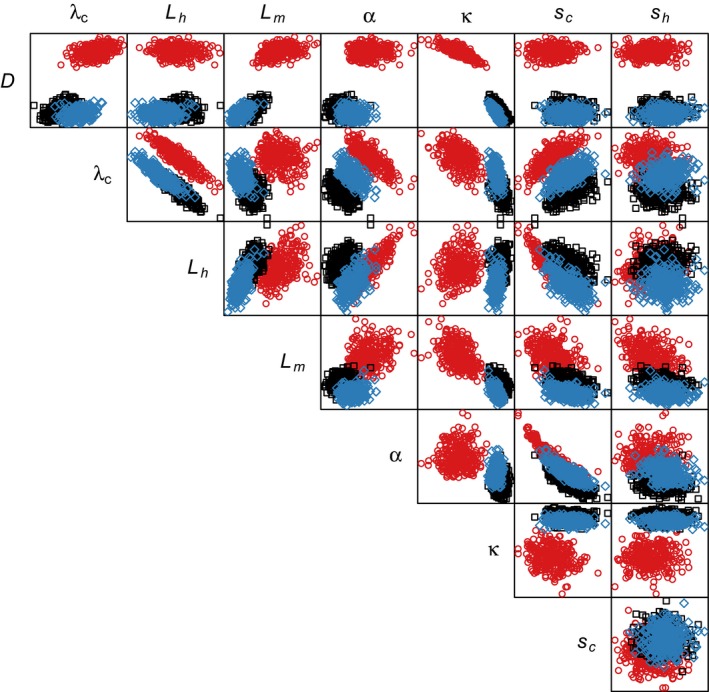
Posterior samples from the MCMC algorithm for all parameters from fits to the data with three sites moved to decrease spatial spread (red circles), the original data (black squares), and the data with two sites moved to increase spatial spread (blue diamonds). See Table 1 for a description of parameters

**Figure 5:**
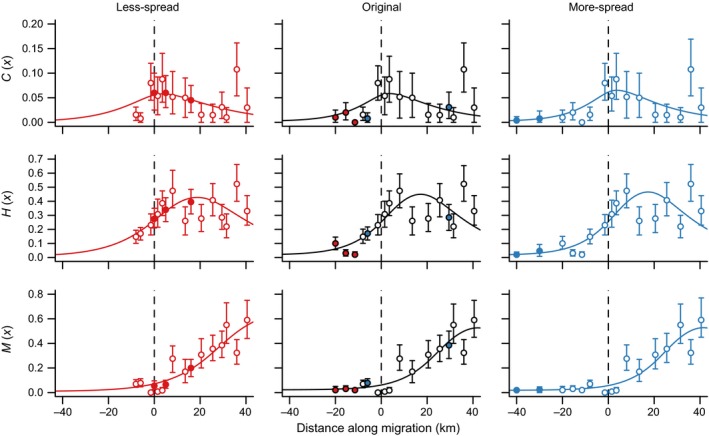
Model fits to the three datasets (red: less spread, black: original data, and blue: more spread), showing the predicted number of copepodid (*C*(*x*)), chalimus (*H*(*x*)), and motile (*M*(*x*)) sea lice per juvenile salmon, used as the expected value in the Poisson likelihood. The data are shown as mean lice per fish ±95% bootstrapped confidence intervals. Solid points in the simulated data are those sites that were added, with the corresponding color in the original data indicating the points that were removed

The parameters controlling the contribution of background and farm sources of sea lice to infections on wild juvenile salmon were nonestimable for the data scenario with less spread in sampling sites; the scaled variance of parameter estimates for κ and α under the less‐spread data scenario did not converge to zero as the number of clones increased (Figure 3). Further, different prior distributions lead to different estimates and standard errors on these parameters (Figure S1). This was particularly true for the ambient source strength, κ (Figure 3a). Both the ambient and farm source strengths became estimable when the original data were used, and these parameters were also estimable for the simulated data with more spatial spread in sample sites (Figure 3a, b and Figure 4). Once again, this was evident from the estimates under different prior distributions, which converged when the spatial spread of the data increased (Figure S2). For the data scenario with increased spatial spread, sites added earlier in the migration route were outside the footprint of the salmon farm, and so they helped only to distinguish the background louse abundance. However, when sites were removed so that the sampling only covered areas affected by the salmon farm, it became more difficult to distinguish farm and ambient sources (Figure 5).

**Figure 6:**
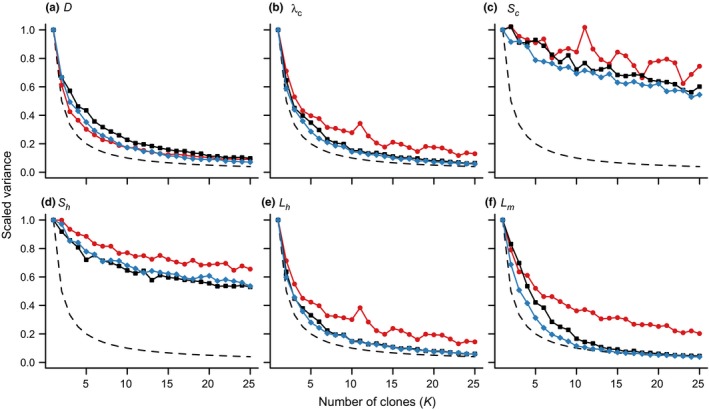
Posterior variance for other model parameters scaled by the variance for a single clone over the number of clones (*K*) for three different data scenarios. The dashed line indicates the ideal rate of decline in scaled variance of 1/*K*. See Figure 3 for the main parameters of interest and key

The survival of sea lice transitioning from copepodid to chalimus stages (*s*_c_) and from chalimus to motile stages (*s*_h_) was nonestimable even as we increased the spatial spread of sampling (Figure 6). Under the more‐spread data scenario, the estimates of survival and standard errors depended on the prior distribution (Figure S3). This may have to do with the relatively low prevalence of copepodid‐stage sea lice, and inconsistent difference in prevalence between chalimus and motile stages (Figure S4).

For all parameters except those for survival *s*_c_ and *s*_h_ (which could not be reliably estimated), the posterior parameter estimates depended on the assumed prior distribution when the model was fit to the less‐spread data (Figure S1). However, when using the original and more‐spread datasets, these estimates converged to the same value when 20 clones were used, regardless of the prior distribution assumed (Figures S2–S3). This also suggests that the parameters of interest were estimable when the spatial spread of the data was increased, and that the estimates are invariant to the choice of prior distribution (Campbell & Lele, 2014).

**Figure 7:**
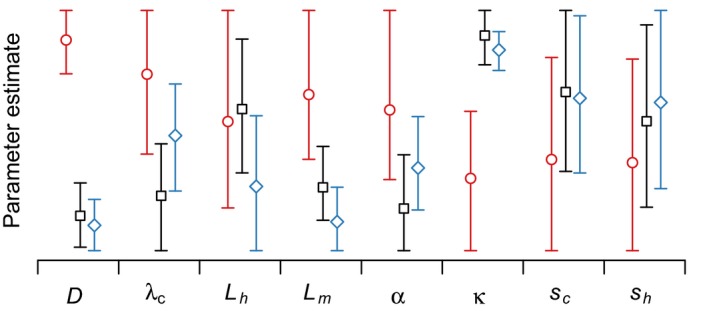
Estimates for the eight free parameters (Table 1) from 10 clones of the data with less spatial spread (red circles), the original data (black squares), and more spatial spread (blue diamonds). The parameters of interest where the estimates differed significantly depending on the spatial spread of the data are *D*, α, and κ. Each parameter is plotted on its own scale (not shown), but the scale of the y‐axis is consistent among the three datasets for the same estimate

The model fits to all three datasets gave very similar predictions for the number of sea lice on juvenile salmon (Figure 5), even though the parameter estimates giving rise to those predictions were not necessarily the same (Figure 7). In particular, the estimates for the diffusion coefficient and the background source strength changed significantly moving from the less‐spread dataset to the original data. Using the less‐spread data, the diffusion coefficient (*D*) was estimated to be much higher and the ambient source strength was estimated to be lower (Figure 7). Because the spatial spread of the data was limited in the scenario with less spatial spread, the ambient source strength was confounded by higher diffusion of farm‐source sea lice.

## 4 Discussion

Model formulation and simulation are key steps in the scientific process that, ideally, should be carried out before data collection in order to inform experimental design. Traditional tools to guide study design include prospective power analyses to determine the sample size required to detect an effect should one exist (Peterman, 1990; Steidl et al., 1997; Toft & Shea, 1983). In an age of increasingly complex, mechanistic models in ecology, more sophisticated tools are needed to ensure that the parameters of interest can be uniquely estimated given the data to be collected. Here, we have presented data cloning (Lele et al., 2007) as a statistical tool that can be used to assess parameter estimability (Lele et al., 2010) for dynamical models and ensure the appropriate spatial and/or temporal scales of sampling in ecological studies.

Collecting more data is often cited as a means to increase statistical power (Peterman, 1990; Steidl et al., 1997) and may in some cases solve problems of parameter nonestimability. However, for models that describe temporal or spatial dynamics, the location of data points in time or space may be more important than the quantity of data. If the model describes a spatial process, collecting additional years of data may not improve parameter estimability if the additional data are collected at the same points in space. Similarly, if the model describes a long‐term cycle in some time series, then collecting data from more individuals or more locations may not make parameters estimable if the data simply do not span a long enough time period to capture the cycle being described.

Our case study involving a mechanistic model for the transmission of sea lice from farmed to wild salmon showed that estimating the relative importance of farm and ambient sources of sea lice required data on sea louse abundance over a 60–80 km corridor centered on the farm location. Initial attempts at quantifying sea louse transmission from farmed salmon assessed infections on wild salmon up to 1 km from the salmon farm—much too small a radius to detect any spatial decline in infection indicative of a point source (M. Krkošek, personal communication). Later studies revealed that sea lice can disperse up to 30 km as free‐living larvae (Krkošek, Lewis, et al., 2005; Krkošek et al., 2006). In this case, the magnitude of currents causing the diffusion of sea lice from point sources at salmon farms was required before simulation analyses could be used to look at parameter estimability under different data collection scenarios. In general, pilot studies may be required to obtain rough estimates of the spatial and/or temporal scale of the process under study before more detailed simulations can be performed to assess parameter estimability.

In cases where some parameters are found to be nonestimable, it may still be possible to draw some inference. First, data cloning can be used to investigate whether combinations of parameters can be estimated (Lele et al., 2010). In some cases, there may be an ecologically relevant function of parameters that is estimable even if the individual parameters themselves are not. Second, nonestimable parameters may not necessarily present a problem if there are not central to the ecological question be asked. In our case, the parameters of interest in our study (i.e., κ and α) were estimable shown to be consistent for different priors, even though survival estimates were not (Figure S3). However, it is not well established that those estimates will remain consistent in the presence of some nonestimable parameters. Although we do not know of any example where some parameters are nonestimable and that makes estimates of other parameters biased, this is not necessarily a generalizable result. Inference for the so‐called partially identified models is an active area of research (e.g., Gustafson, 2015; Romano & Shaikh, 2008). Thus, caution should be exercised when drawing inference from models where some parameters are shown to be nonestimable.

Obtaining additional data or altering the study design is not always possible. For example, long‐term monitoring data are often collected without a particular hypothesis in mind and may be subsequently used in many different studies. In these cases, parameter nonestimability cannot be addressed by collecting new data or altering the study design, but data from other sources may make parameters estimable. In an extension of the sea louse transmission model with multiple farm sources (Krkošek et al., 2006), the number of sea lice on each salmon farm from industry data can be used to constrain the relative strengths of the different sources. This additional constraint, although it may seem like added complexity, made the farm‐source strengths estimable (Peacock et al. in prep.).

## 5 Conclusions

Mechanistic models can improve the inferences made from noisy and sparse ecological data, but the potential for parameter nonestimability when fitting such models is too‐often ignored (Lele, 2010). Often, collecting *more* data, which usually requires additional resources, may not be required to solve estimability problems; it may be that collecting *different* data is all that is needed. Data cloning is a new statistical tool that can be used to assess parameter estimability during data analysis stage (Lele et al., 2007, 2010), but may also be useful in designing studies with the appropriate spatial and/or temporal scales of sampling to ensure that parameters of interest will be estimable. Our case study highlighted that assessing parameter estimability should be a key step in study design where fitting complex mechanistic models is the end goal.

## Conflict of interest

None declared.

## Data accessibility

Data and R Code for the analyses can be found at https://github.com/sjpeacock/DataCloning4StudyDesign.
