# Supplementary material for: Study design and parameter estimability for spatial and temporal ecological models
Source: Ecol Evol. 2016 Dec 30;7(2):762–70. doi: 10.1002/ece3.2618 (PMC5243787; doi:10.1002/ece3.2618)
Supplement: Supplementary file 1 [file ECE3-7-762-s001.pdf]

Study design and parameter estimability for spatial and temporal ecological models

Stephanie Jane Peacock[1,2,*,†], Martin Krkošek[1,3], Mark Alun Lewis[2,4] and Subhash Lele[4]

1. Ecology and Evolutionary Biology, University of Toronto, Toronto ON M5S 3B2
2. Biological Sciences, University of Alberta, Edmonton AB T6G 2G1
3. Salmon Coast Field Station, Simoom Sound, BC V0P 1S0
4. Mathematical and Statistical Sciences, University of Alberta, Edmonton AB T6G 2G1

* Current address: Biological Sciences, University of Calgary, Calgary AB T2N 1N4
† Corresponding author: stephanie.j.peacock@gmail.com

Supporting information

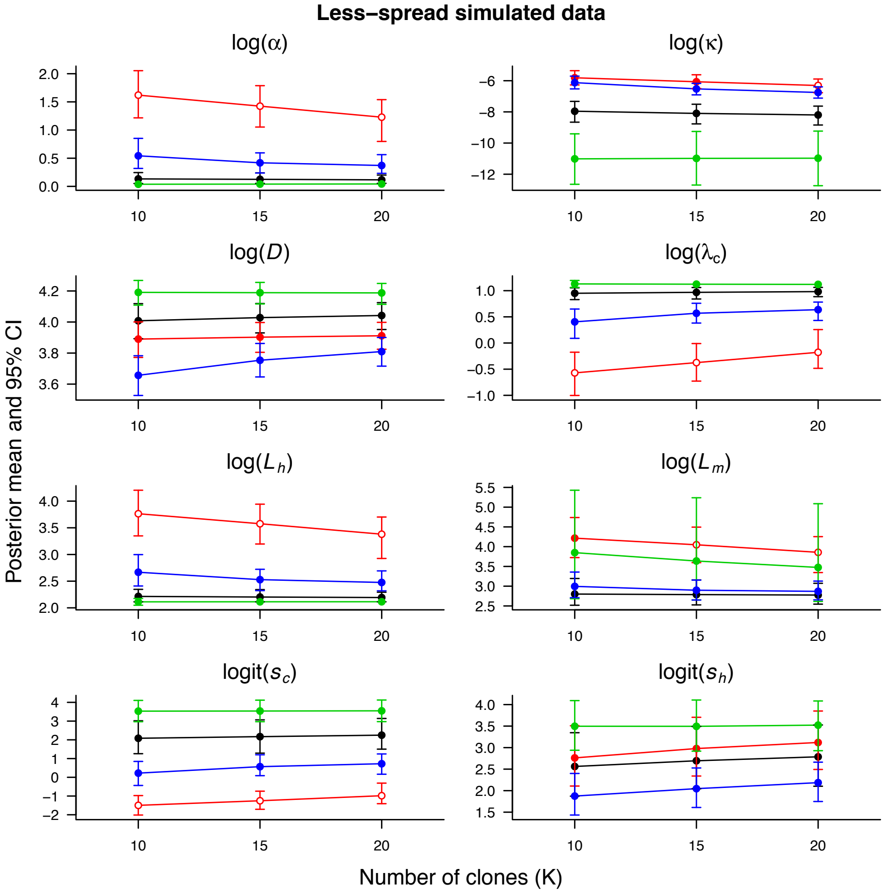

Figure S 1. Posterior estimates and 95% credible intervals using 10, 15, and 20 clones of the less-spread simulated data, under four different prior assumptions (black = prior 1, red = prior 2, green = prior 3, and blue = prior 4; Table S1). The two parameters of interest, log(α) and log(κ), do not appear to converge to the same estimate under the different prior assumptions, indicating non-estimability in this case (Campbell & Lele 2014). Open points (e.g., in log(α)) are those runs where the MCMC algorithm did not converge (i.e., $\hat{R} > 1.1$).

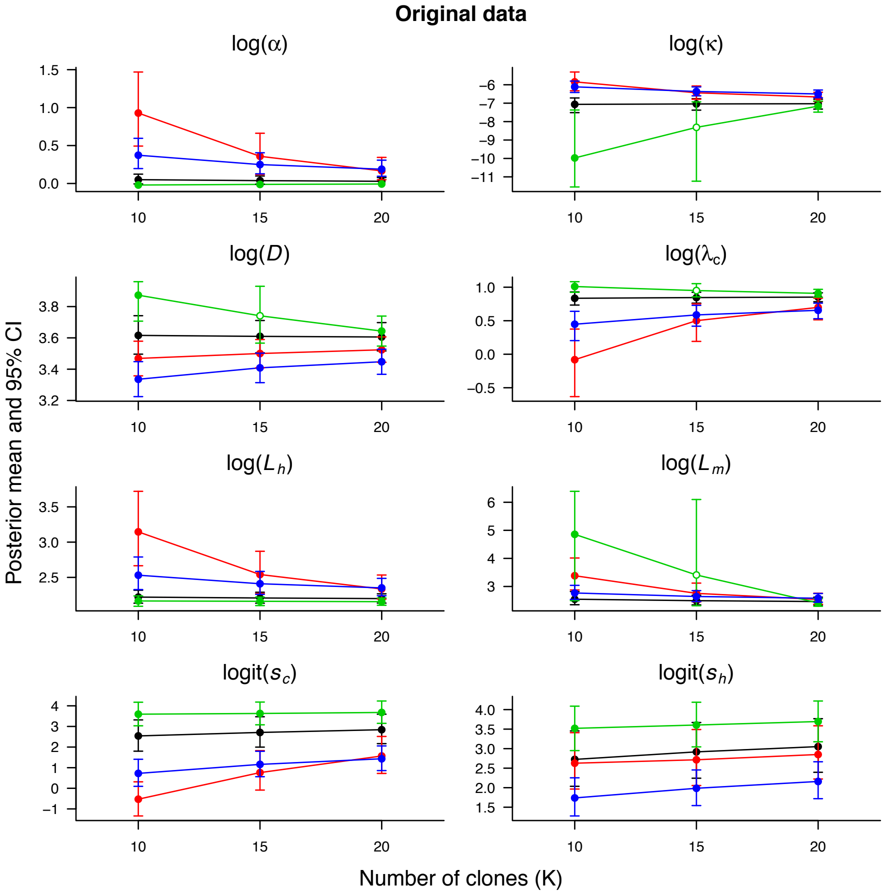

Figure S 2. Posterior estimates and 95% credible intervals using 10, 15, and 20 clones of the original data, under four different prior assumptions (black = prior 1, red = prior 2, green = prior 3, and blue = prior 4; Table S1). The two parameters of interest, log(α) and log(κ), are converging to the same estimate under all prior assumptions, indicating estimability in this case (Campbell & Lele 2014). However, other parameter estimates appear to depend on the prior distribution (e.g., logit(s_h)). Open points are those runs where the MCMC algorithm did not converge (i.e., $\hat{R} > 1.1$).

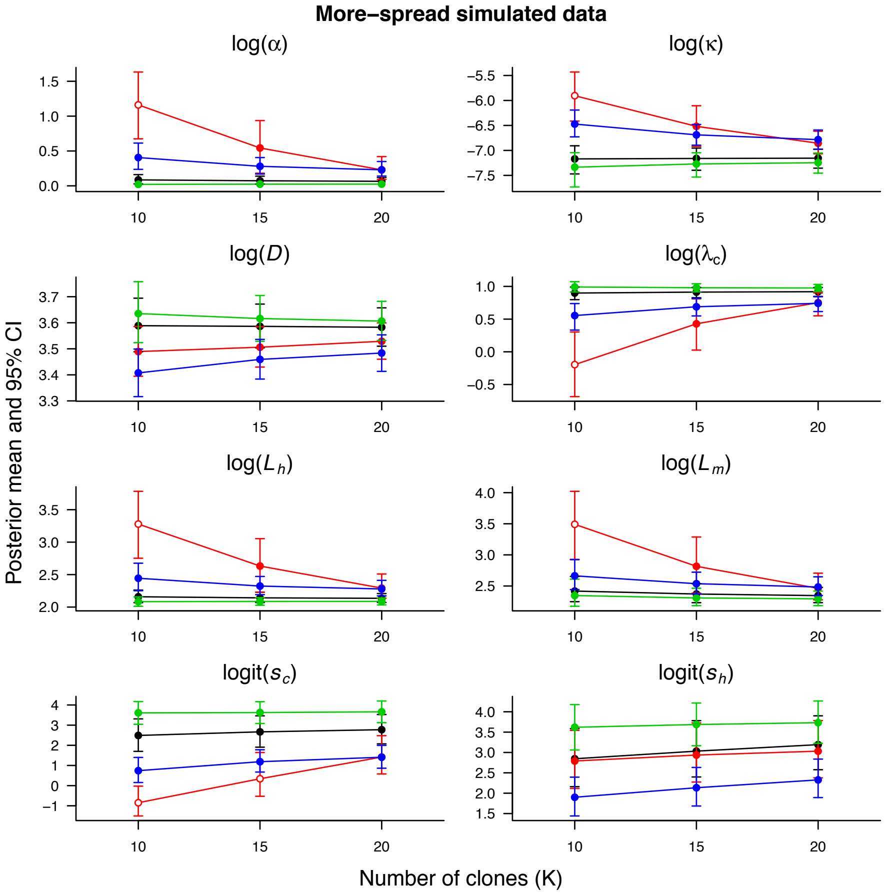

Figure S 3. Posterior estimates and 95% credible intervals using 10, 15, and 20 clones of the more-spread simulated data, under four different prior assumptions (black = prior 1, red = prior 2, green = prior 3, and blue = prior 4; Table S1). The two parameters of interest, log(α) and log(κ), are converging to the same estimate under all prior assumptions, indicating estimability in this case (Campbell & Lele 2014). Open points are those runs where the MCMC algorithm did not converge (i.e., $\hat{R} > 1.1$).

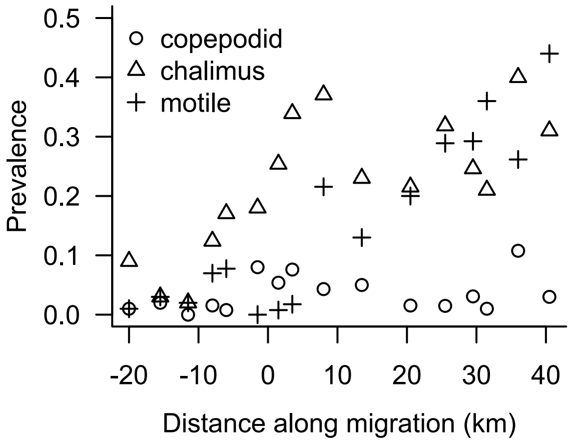

Figure S 4. The prevalence (proportion of salmon in the sample infected with at least one sea louse) of copepodid-, chalimus- and motile-stage lice throughout the migration.

Table S 1. Different prior assumptions for parameters shown in Figures S2-S4.

Parameter	Description	Prior 1	Prior 2	Prior 3	Prior 4
D	Diffusion coefficient in dispersal from farm point source	$\mu = 3.5$<br>$\sigma = 0.5$	$\mu = 5.0$<br>$\sigma = 0.3$	$\mu = 2.0$<br>$\sigma = 1.0$	$\mu = 0$<br>$\sigma = 0.5$
$\kappa\beta v^{-1}$	Strength of background sources	$\mu = -7.0$<br>$\sigma = 0.5$	$\mu = -5.0$<br>$\sigma = 0.3$	$\mu = -10$<br>$\sigma = 1.0$	$\mu = -2.0$<br>$\sigma = 0.5$
$\alpha\beta v^{-1}$	Strength of farm source	$\mu = 0$<br>$\sigma = 0.5$	$\mu = 4.0$<br>$\sigma = 0.3$	$\mu = -3.0$<br>$\sigma = 1.2$	$\mu = 2.0$<br>$\sigma = 0.5$
s_c, s_h	Survival of copepodid and chalimus stage lice to the next stage	$\mu = 2.0$<br>$\sigma = 0.5$	$\mu = 0$<br>$\sigma = 0.7$	$\mu = 3.5$<br>$\sigma = 0.3$	$\mu = -1.0$<br>$\sigma = 0.5$
λ_c	Distance travelled by juvenile salmon during the duration of the copepodid stage	$\mu = 1.0$<br>$\sigma = 0.5$	$\mu = 5.0$<br>$\sigma = 1.5$	$\mu = 2.5$<br>$\sigma = 0.3$	$\mu = -1.0$<br>$\sigma = 0.5$
$L_h = \lambda_h / \lambda_c$<br>$L_m = \lambda_m / \lambda_c$	The distances traveled during cumulative time for development of lice to chalimus and motile stages, relative to λ_c	$\mu = 3.0$<br>$\sigma = 0.5$	$\mu = 1.2$<br>$\sigma = 0.5$	$\mu = 5.0$<br>$\sigma = 1.0$	$\mu = 0$<br>$\sigma = 0.5$

* Priors are on log-scale for all parameters except s_c, s_h which are on the logit-scale.
